# Microarray Analysis Reveals Increased Transcriptional Repression and Reduced Metabolic Activity but Not Major Changes in the Core Apoptotic Machinery during Maturation of Sympathetic Neurons

**DOI:** 10.3389/fncel.2016.00066

**Published:** 2016-03-16

**Authors:** Mikk Raba, Jaan Palgi, Maria Lehtivaara, Urmas Arumäe

**Affiliations:** ^1^Department of Gene Technology, Tallinn University of TechnologyTallinn, Estonia; ^2^Biomedicum Functional Genomics Unit, Biomedicum Helsinki, University of HelsinkiHelsinki, Finland; ^3^Institute of Biotechnology, University of HelsinkiHelsinki, Finland

**Keywords:** sympathetic neurons, maturation, microarray, programmed cell death, nerve growth factor

## Abstract

Postnatal maturation of the neurons whose main phenotype and basic synaptic contacts are already established includes neuronal growth, refinement of synaptic contacts, final steps of differentiation, programmed cell death period (PCD) etc. In the sympathetic neurons, postnatal maturation includes permanent end of the PCD that occurs with the same time schedule *in vivo* and *in vitro* suggesting that the process could be genetically determined. Also many other changes in the neuronal maturation could be permanent and thus based on stable changes in the genome expression. However, postnatal maturation of the neurons is poorly studied. Here we compared the gene expression profiles of immature and mature sympathetic neurons using Affymetrix microarray assay. We found 1310 significantly up-regulated and 1151 significantly down-regulated genes in the mature neurons. Gene ontology analysis reveals up-regulation of genes related to neuronal differentiation, chromatin and epigenetic changes, extracellular factors and their receptors, and cell adhesion, whereas many down-regulated genes were related to metabolic and biosynthetic processes. We show that termination of PCD is not related to major changes in the expression of classical genes for apoptosis or cell survival. Our dataset is deposited to the ArrayExpress database and is a valuable source to select candidate genes in the studies of neuronal maturation. As an example, we studied the changes in the expression of selected genes *Igf2bp3, Coro1A, Zfp57, Dcx*, and *Apaf1* in the young and mature sympathetic ganglia by quantitative PCR and show that these were strongly downregulated in the mature ganglia.

## Introduction

Maturation of the neurons occurs when the differentiation is mainly completed and the contacts with synaptic targets are mostly established. It includes enlargement of cell soma, strengthening of cytoskeleton, elaboration and refinement of synaptic contacts and other final steps of differentiation. A remarkable event in the maturating neuronal populations is the programmed cell death (PCD) period, where many neurons die apoptotically, thereby determining the final number of neurons (Oppenheim, 1991). In the neurons that project out of the brain, such as sensory, autonomic and spinal motoneurons the final number is determined by the extent of trophic support provided by target-derived neurotrophic factors (NTFs) that rescue the neurons from apoptosis (Bibel and Barde, 2000; Huang and Reichardt, 2001). The PCD lasts during a relatively short time period and when it ends, the neurons are not apoptotic anymore and do not require trophic support for survival. Compared to the processes of the early neurogenesis, including the cell fate determination, migration, and early differentiation of the neurons during embryogenesis, the postnatal maturation of neurons is poorly studied.

Sympathetic neurons of the superior cervical ganglion (SCG) are one of the best studied models for neuronal development. In mouse, the neural crest-derived precursors form the SCG primordia between embryonic day (E) 11.5–14.5 (Nishino et al., 1999). Most of SCG neurons become postmitotic before birth of the animals (Hendry, 1977; Shi et al., 2008). The majority of SCG neurons are noradrenergic and the main neurotransmitter-synthesizing enzymes tyrosine hydroxylase and dopamine β hydroxylase are expressed already in the early sympathetic precursors (Pattyn et al., 1999). The neuritogenesis, target innervation and synaptogenesis of the sympathetic neurons begin in embryogenesis and continue after the birth (De Champlain et al., 1970; Schotzinger and Landis, 1990). During postnatal maturation, the somata of SCG neurons enlarge and synaptic contacts with the targets are refined. The PCD period in SCG neurons occurs during first two postnatal weeks when about 30% of the neurons die (Wright et al., 1983). Nerve growth factor (NGF), secreted by the targets of innervation in the limited amounts, rescues the neurons that get it (Harrington and Ginty, 2013) whereas neurons that remain without NGF die by unsuppressed apoptosis (Chang et al., 2002; Putcha and Johnson, 2004; Kristiansen et al., 2011; Kristiansen and Ham, 2014). The PCD of the SCG neurons ends by about second postnatal week and the neurons are then not dying any more in the absence of NGF (Kole et al., 2013). How the NGF-dependency disappears during maturation of SCG neurons is not known but it occurs with the same time schedule both *in vivo* and in the cultured neurons (Easton et al., 1997; Francis and Landis, 1999; Putcha et al., 2000; Glebova and Ginty, 2005; Young et al., 2011) and remains then a persistent trait, obviously via changes in genome activity. Such stable changes in gene expression could most probably be the basis for many other maturational events as well. However, while the early specification and differentiation of sympathetic neurons are extensively studied (Apostolova and Dechant, 2009; Cane and Anderson, 2009; Young et al., 2011), the postnatal maturation has deserved much less attention (Glebova and Ginty, 2005; Kole et al., 2013).

Here we set up on search for significantly changed genes during maturation of the SCG neurons. We compared by microarray gene expression profile of the immature (5 DIV—days *in vitro*) and mature (21 DIV) cultures of SCG neurons from newborn mice. Our dataset is deposited to the ArrayExpress database and is a valuable source to select candidate genes in the studies of neuronal maturation. As an example, we studied the changes in the expression of five genes that could be related to neuronal maturation, by quantitative PCR (qPCR) on the whole immature and mature SCG ganglia of mice and rats.

## Materials and methods

### Neuronal cultures

**Figure 1:**
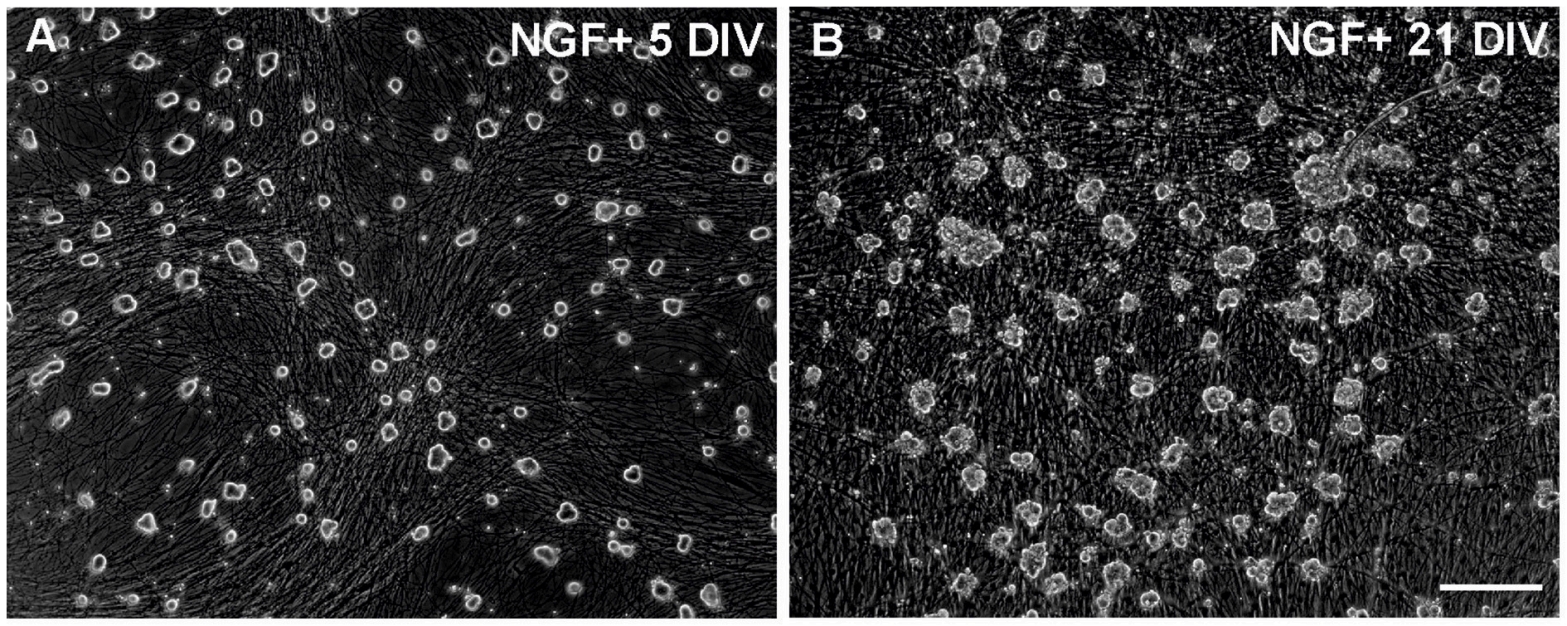
**Neuronal cultures used for the microarray study. (A,B)** Images of typical 5 DIV and 21 DIV cultures of the SCG neurons taken before cell collection. The 21 DIV cultures contain many non-neuronal cells but most of them remained attached to the glass surface after collection of the neurons. Scale bar, 100 μM.

All procedures for animal use were approved by the Estonian Ethics Committee and University of Helsinki Laboratory Animal Centre (Protocol number KEK11-020). SCG neurons from newborn NMRI mice were prepared as described earlier (Yu et al., 2003; Hellman et al., 2011; Jakobson et al., 2012, 2013). The neurons were plated onto glass coverslips coated with type IV collagen (BD Biosciences, San Diego, CA, USA, #354233) and grown for 5 DIV (immature) or 21 DIV (mature) in the Neurobasal medium (Invitrogen Ltd, UK) containing B27 serum substitute (Invitrogen) and 30 ng/ml of NGF (PeproTech, Rocky Hill, NJ, USA; #450-01). The culture medium was changed twice a week. The neurons were harvested in phosphate-buffered saline, pelleted and dissolved in the RNeasy RLT lysis buffer (Qiagen, Venlo, Netherlands, #79216). Six independent cultures for mature neurons and three independent cultures for immature neurons (ganglia from 12 to 17 pups per culture) were prepared. The majority of the non-neuronal cells were removed by pre-plating step (Yu et al., 2003; Hellman et al., 2011; Jakobson et al., 2012, 2013) but some always remained and proliferated. The majority of these non-neuronal cells remained attached to the glass coverslips after collection of the cells for RNA extraction. However, to exclude genes of the non-neuronal cells from the analysis with certainty we also collected non-neuronal cells without neurons from the preplating step, that were grown on the non-coated dishes in Neurobasal/B27 medium without NGF for 5 DIV and 21 DIV, one sample for both. No neurons remained alive in these conditions. To verify that the neurons had indeed matured *in vitro* we tested whether the 21 DIV neurons have become independent of NGF for survival, a clear hallmark of the mature SCG neurons. The 5 DIV or 21 DIV cultures were washed three times with NGF-free medium and function-blocking anti-NGF antibodies (Chemicon MAB5260Z) were added. For comparison, the neurons were washed and re-supplemented with NGF. The original number of living neurons was counted under the inverted microscope immediately after NGF deprivation and again 5 days later. The experiment was repeated two times. On all repeats, all 21 DIV neurons remained alive for 5 additional DIV without or with NGF, as reported by others (Easton et al., 1997; Putcha et al., 2000) whereas the 5 DIV neurons died by NGF deprivation as predicted (Yu et al., 2003; Hellman et al., 2011; Mätlik et al., 2015) (not shown). Typical images are shown on Figure 1. The images were captured with inverted microscope (model DM-IRB; Leica, Germany) using HC PL FLUOTAR objective (10 × /0.30), and a 3CCD color video camera (model DXC-950P; Sony) under the control of Image-Pro Plus software version 3.0 (Media Cybernetics, Inc., Rockville, MD, USA).

### Array hybridization

RNeasy Kit (Qiagen) was utilized for the purification of total RNA. Cells were disrupted with lysis buffer and homogenized passing the lysate through 20-gauge needle. 70% ethanol was added and the sample was applied to an RNeasy spin column. The RNA bound the membrane of the column and the contaminants were washed away. Purified RNA was eluted in the water. Sample RNAs were analyzed for integrity and quality on Agilent Bioanalyzer 2100 (Agilent Technologies, Santa Clara, CA, USA). The labeling and hybridization were done using the Ambion WT Expression Kit (Ambion, Austin, TX, USA) and Affymetrix GeneChip WT Terminal Labeling Kit (Affymetrix, Santa Clara, CA, USA), following the manufacturer's instructions. The starting amount of total RNA was 50 ng. 15 μg of cRNA was used for single-stranded cDNA-synthesis. A total of 5.5 μg of single-stranded cDNA was fragmented and hybridized onto a Mouse Exon 1.0 ST Array (Affymetrix, Santa Clara, CA, USA) during 17-h incubation at 45°C. Immediately following hybridization, the array was processed using an automated protocol on the GeneChip® Fluidics Station 450, followed by scanning on a GeneChip® Scanner.

### Microarray data analysis

Signal values were analyzed using Bioconductor (R). Re-mapped gene annotations from the Brainarray Custom chip description files were used (MoEx10stv1_Mm_ENSG, v.17.1.0, http://brainarray.mbni.med.umich.edu/Brainarray/), and additional gene information was extracted from EnsEMBL using Biomart-package. Robust Multichip Average (RMA) was used for background correction, quantile normalization and summarization of the signal values. Gene expression values were compared between the immature and mature neurons using the moderated *t*-statistic of limma-package. Benjamini-Hochberg (FDR) procedure was used for multiple testing correction. Genes with FDR adjusted *p* < 0.05 and fold-change >2 were considered significantly differentially expressed. List of these significantly up- and down-regulated genes were used for gene ontology enrichment analysis, which was performed with web-based DAVID software (http://david.abcc.ncifcrf.gov/) (Huang da et al., 2007).

### Real-time quantitative PCR analysis

RNA from freshly prepared newborn, 14 days old and 21 days old mouse and Sprague Dawley rat SCG, as well as from total brain was purified with GeneElute Mammalian Total RNA Miniprep kit (Sigma, St. Louis, MO, USA). DNase treatment was done separately. RNA clean-up was done with the same RNA purification kit. cDNA synthesis was performed in 20 μl reaction containing 4 μl of 5x RT buffer (Solis Biodyne, Tartu, Estonia), 2.5 μl of 2.5 mM mix of 4 dNTPs (Solis BioDyne), 1 μl of Oligo (dT)_18_ primer (Thermo Fisher Scientific, Rockford, IL, USA), 0.2 μl of Random Hexamer Primer (Thermo Fisher Scientific), 0.5 μl of RiboLock RNase Inhibitor (Thermo Fisher Scientific), 0.5 μl of 200 U/μl MMLV Revertase RNase H minus (Solis BioDyne) and 2 μg of RNA in 11.3 μl water. cDNA was synthesized at 37°C for 90 min and kept thereafter for 5 min at 95°C to inactivate the polymerase. The whole reaction mixture was diluted 2x with RNase-free water to standardize the cDNA amount as 1 μg of RNA in 20 μl of cDNA synthesis reaction. The quality of cDNA synthesis was estimated by PCR for *cyclophilin G* expression.

qPCR analysis was done using Roche Light Cycler 480 II equipment (Roche, Penzberg, Germany). Ten μl reaction mixture contained 5 μl of LightCycler 480 SYBR Green I Master mix (Roche), 10 μM forward and reverse primers (0.5 μl each), 0.8 μl of cDNA (RT mix) and the rest of the volume constituted with RNase-free water. Each reaction was run in triplicates and includes the enzyme activation step 95°C for 4 min continued for 40 times with the following steps: 95°C for 10 s, 56°C for 10 s, 72°C for 10 s. Each run was finished with the melting curve. Neuron-specific Neurofilament H (*Nefh*) was used as the endogenous control gene. *Nefh* appears over threshold at Cp 24–25 in our qPCR conditions in all runs. The laboratory golden standard was the 1:1 mixture of RNAs prepared from the newborn and 21 day whole brains. This mixture, representing all the genes analyzed by qPCR, was used as calibrator in every run. Primer sequences are listed in Supplementary file S2. Three or four independent experiments were performed. In each repeat, all genes were analyzed simultaneously on the same PCR run. The means were statistically analyzed with one-way ANOVA followed by Tukey-Kramer Multiple Comparisons Test using GraphPad InStat 3 program (GraphPad Software, Inc., CA, USA).

## Results

### Microarray profiling of gene expression in immature and mature sympathetic neurons

**Figure 2:**
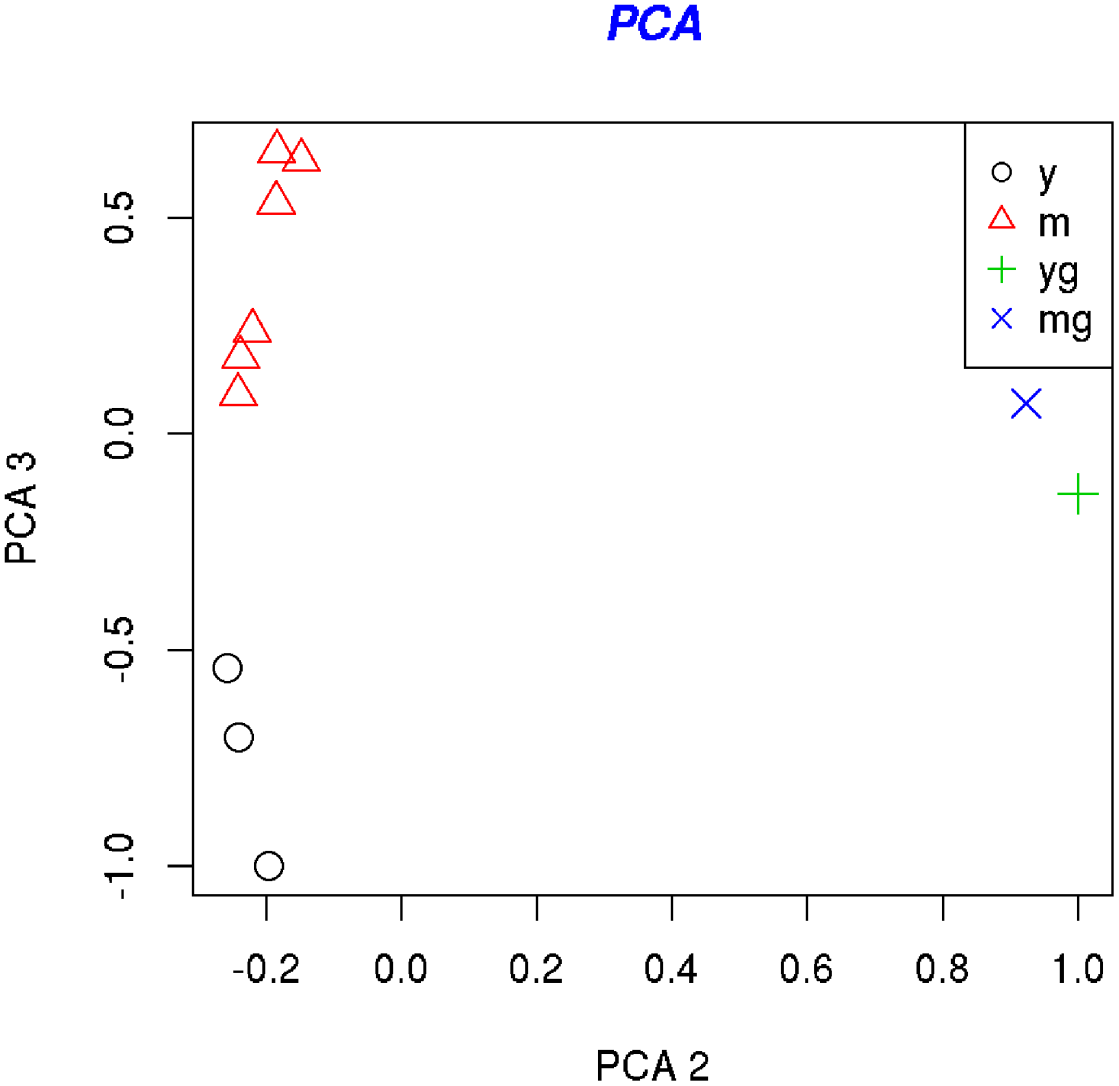
**PCA analysis of the samples**. PCA-plots show the location of the samples along the first and second principle component (left) and along the second and third principle components (right). The second principal component separates young (5 DIV) (yg) and mature (21 DIV) (mg) glial cell samples from the neuronal samples, whereas the third principal component separates the young (5 DIV) (y) and mature (21 DIV) (m) neuronal samples.

To reveal the genes that are differently expressed in the immature and mature SCG neurons the biotin-labeled single-stranded cDNA samples were hybridized to the Affymetrix GeneChips® Mouse Exon 1.0 ST microarrays. To assess the quality of data, all experimental groups: immature 5 DIV neurons (three repeats) mature 21 DIV neurons (six repeats), 5 DIV non-neuronal cells (one repeat) and 21 DIV non-neuronal cells (one repeat) were subjected to principle component analysis (PCA) and hierarchical clustering. The PCA-plot (Figure 2) shows that immature neurons, mature neurons and non-neuronal cells cluster separately from each other but the repeats within these groups cluster together. No obvious outliers were detected. The same conclusions were drawn from hierarchical clustering (not shown).

The significance of differential expression between 5 DIV and 21 DIV neuronal samples, as well as between 5 DIV and 21 DIV non-neuronal samples was assessed using the empirical Bayes moderated *t*-statistics followed by *p*-value adjustment with false discovery rate (FDR) approach. Importantly, the genes that changed significantly in the non-neuronal cells during 3-week culture almost completely differed from those that changed during 3-week maturation of neurons (not shown). We therefore conclude that the small number of non-neuronal cells remaining in the neuronal samples after cell collection could not significantly affect the interpretation of array data.

In the neuronal samples, of about 26,800 genes analyzed, 2461 changed with adjusted *p* < 0.05, 1310 of these being up-regulated and 1151 down-regulated. Table 1 shows the number of differentially expressed genes with increasingly stringent cut-off parameters. 39 genes were up-regulated and 30 genes down-regulated more than log 1.5-fold with adjusted *p* < 0.01. These genes are listed in the Tables 2, 3, respectively. Data of the whole array are available from the ArrayExpress database (http://www.ebi.ac.uk/arrayexpress) under accession number E-MTAB-3221.

**Table 1:** **Number of significantly up-or down-regulated genes using different adjusted *p*-values and log fold changes (logFC)**.

	**Up**	**Down**
adj *p* < 0.05	1310	1151
adj *p* < 0.01	587	461
adj *p* < 0.05, logFC >1	169	124
adj *p* < 0.01, logFC >1	121	94
adj *p* < 0.05, logFC >1.5	58	33
adj *p* < 0.01, logFC >1.5	39	30
adj *p* < 0.05, logFC >2	18	11
adj *p* < 0.01, logFC >2	10	10

**Table 2:** **Most significantly up-regulated genes in the 21 DIV compared to 5 DIV SCG neurons (adjusted *p* < 0.01, log fold change >1.5)**.

**Gene ID**	**Description**	**log FC**	**adj *p*-value**
Gjc3	Gap junction protein, gamma 3	−3.24	0.011109
Lynx1	Ly6/neurotoxin 1	−3.22	0.000001
Cdk18	Cyclin-dependent kinase 18	−2.79	0.000605
Wls	Wntless homolog (Drosophila)	−2.39	0.006118
Ajap1	Adherens junction associated protein 1	−2.33	0.013279
Gda	Guanine deaminase	−2.31	0.000655
Tnfrsf10b	Tumor necrosis factor receptor superfamily, member 10b	−2.29	0.000126
Gria3	Glutamate receptor, ionotropic, AMPA3 (alpha 3)	−2.27	0.000590
Tnr	Tenascin R	−2.25	0.001291
Vwa5a	Von Willebrand factor A domain containing 5A	−2.16	0.000297
Nt5c1a	5′-nucleotidase, cytosolic IA	−2.13	0.000883
Cd44	CD44 antigen	−2.11	0.007235
Cmbl	Carboxymethylenebutenolidase-like (Pseudomonas)	−2.00	0.000186
Mfge8	Milk fat globule-EGF factor 8 protein	−1.99	0.003712
Ano3	Anoctamin 3	−1.99	0.001612
Mybl1	Myeloblastosis oncogene-like 1	−1.98	0.000186
Herc6	Hect domain and RLD 6	−1.97	0.000264
Cast	Calpastatin	−1.97	0.000186
Ly96	Lymphocyte antigen 96	−1.97	0.000186
Wbscr27	Williams Beuren syndrome chromosome region 27 (human)	−1.93	0.001612
Ctla2a	Cytotoxic T lymphocyte-associated protein 2 alpha	−1.90	0.008117
Ccnd2	Cyclin D2	−1.90	0.011249
1700007K13Rik	RIKEN cDNA 1700007K13 gene	−1.89	0.000034
Bmp7	Bone morphogenetic protein 7	−1.84	0.004110
Gal	Galanin	−1.84	0.001046
Crebrf	CREB3 regulatory factor	−1.82	0.003056
Susd2	Sushi domain containing 2	−1.82	0.001636
Hcn1	Hyperpolarization-activated, cyclic nucleotide-gated K+ 1	−1.80	0.002706
Ell2	Elongation factor RNA polymerase II 2	−1.80	0.001770
Stard13	StAR-related lipid transfer (START) domain containing 13	−1.73	0.011192
Sh3bgrl2	SH3 domain binding glutamic acid-rich protein like 2	−1.73	0.000340
Eda2r	Ectodysplasin A2 receptor	−1.72	0.002128
Ptprm	Protein tyrosine phosphatase, receptor type, M	−1.71	0.009108
Ephx1	Epoxide hydrolase 1, microsomal	−1.69	0.000208
Slc41a3	Solute carrier family 41, member 3	−1.66	0.005057
Frmpd4	FERM and PDZ domain containing 4	−1.65	0.003490
Itgb5	Integrin beta 5	−1.64	0.000883
Sulf2	Sulfatase 2	−1.62	0.003117
Mr1	Major histocompatibility complex, class I-related	−1.59	0.008301
Gbp7	Guanylate binding protein 7	−1.58	0.000330
Slc22a18	Solute carrier family 22 (organic cation transporter), member 18	−1.57	0.004334
Hist1h2bc	Histone cluster 1, H2bc	−1.56	0.003142
Rasd2	RASD family, member 2	−1.54	0.000838

**Table 3:** **Most significantly down-regulated genes in the 21 DIV compared to 5 DIV SCG neurons (adjusted *p* < 0.01, log fold change >1.5)**.

**Gene ID**	**Description**	**log FC**	**adj *p*-value**
Slc38a4	Solute carrier family 38, member 4	4.02	0.000186
Agtr2	Angiotensin II receptor, type 2	3.34	0.000126
Dchs1	Dachsous 1 (Drosophila)	3.12	0.000034
Car2	Carbonic anhydrase 2	2.77	0.000007
Coch	Coagulation factor C homolog (Limulus polyphemus)	2.64	0.001140
Shisa6	Shisa homolog 6 (Xenopus laevis)	2.47	0.000264
Crtac1	Cartilage acidic protein 1	2.32	0.001345
Stk32b	Serine/threonine kinase 32B	2.19	0.000932
Slc16a12	Solute carrier family 16 (monocarboxylic acid transporters), member 12	2.13	0.004841
Dcx	Doublecortin	2.10	0.000497
Dpp10	Dipeptidylpeptidase 10	2.06	0.014455
B3gnt5	UDP-GlcNAc:betaGal beta-1,3-N-acetylglucosaminyltransferase 5	1.96	0.000374
Zfp57	Zinc finger protein 57	1.94	0.000649
Vwa5b2	Von Willebrand factor A domain containing 5B2	1.84	0.000264
Igf2bp3	Insulin-like growth factor 2 mRNA binding protein 3	1.81	0.000828
4833424O15Rik	RIKEN cDNA 4833424O15 gene	1.72	0.000520
Gpr119	G-protein coupled receptor 119	1.72	0.000420
BC068157	cDNA sequence BC068157	1.71	0.000186
Gjd2	Gap junction protein, delta 2	1.70	0.013462
St8sia2	ST8 alpha-N-acetyl-neuraminide alpha-2,8-sialyltransferase 2	1.69	0.001166
Fat3	FAT tumor suppressor homolog 3 (Drosophila)	1.68	0.000186
Nxph4	Neurexophilin 4	1.68	0.000066
Rimklb	Ribosomal modification protein rimK-like family member B	1.67	0.001005
4931428F04Rik	RIKEN cDNA 4931428F04 gene	1.64	0.014154
Cacng4	Calcium channel, voltage-dependent, gamma subunit 4	1.62	0.000186
Peg10	Paternally expressed 10	1.62	0.002032
Crip1	Cysteine-rich protein 1 (intestinal)	1.59	0.001874
Cxadr	Coxsackie virus and adenovirus receptor	1.58	0.000208
Efnb3	Ephrin B3	1.58	0.000186
Trim67	Tripartite motif-containing 67	1.57	0.003034
Enpp2	Ectonucleotide pyrophosphatase/phosphodiesterase 2	1.57	0.000355
Efcab3	EF-hand calcium binding domain 3	1.54	0.002390
Asic1	Acid-sensing (proton-gated) ion channel 1	1.50	0.001672

There were no significant changes in the genes for classical markers of sympathetic neurons such as *th* (tyrosine hydroxylase), *ddc* (dopa decarboxylase), *dbh* (dopamine beta hydroxylase), *npy* (neuropeptide Y), *Eno2* (neuron-specific enolase), and *Slc18a2* (vesicular monoamine transporter 2). We conclude that the main sympathetic phenotype is already developed by the first postnatal week.

### Gene ontology analysis

To reveal the potential functional trends in our data, differentially expressed genes with adjusted *p* > 0.05 were associated with Gene Ontology (GO) terms using web-based DAVID software (http://david.abcc.ncifcrf.gov/) (Huang da et al., 2007). Up- and down-regulated genes were analyzed separately. The most abundantly enriched GO-terms among up- and down-regulated genes are presented on Tables 4, 5, respectively. The full cluster analysis is presented in the Supplementary file S1. Of note, many significantly up-regulated GO-terms were related to the neurons, confirming purity of our neuronal samples. As shown in Table 4, the most potently up-regulated GO-terms during SCG neuronal maturation were associated with neuronal differentiation, including the neurites, synapses, ion channels, neurotransmitters etc.

**Table 4:** **Most significantly enriched GO-terms from the genes up-regulated during maturation of the SCG neurons**.

**GO-term**	**F.E**.	***p***	**Genes**
Protein retention in ER lumen (GO:0006621, BP)	11.4	0.02	Kdelr2, Grik5, Kdelr1
Mitotic chromosome condensation (GO:0007076, BP)	8.2	0.05	Akap8, Cdca5, Atf6b
Dendrite morphogenesis (GO:0048813, BP)	7.9	0.003	Dscam, Cacna1a, Ctnna2, Dcx, Slc11a2
Diacylglycerol metabolic process (GO:0046339, BP)	7.6	0.01	Dgkk, Lipe, Agpat6, Dgkd
Long-term memory (GO:0007616, BP)	7.1	0.06	Chst10, Gria1, Grin1
Ephrin receptor activity (GO:0005003, MF)	6.7	0.005	Ephb2, Epha7, Epha10, Epha6, Epha8
Dendritic shaft (GO:0043198, CC)	6.6	0.005	Cacna1b, Psen1, Asic1, Cacna1c, Slc1a2
Voltage-gated calcium channel activity (GO:0005245, MF)	6.2	6.3E-4	Cacna1b, Cacna1a, Cacna1d, Cacng4, Cacna1i, Cacna1c, Ryr1
UDP-galactosyltransferase activity (GO:0035250, MF)	6.2	0.002	B4galt2, Ugt8a, B4galt3, B3galnt1, B3galt1, B3gat1
Retinal ganglion cell axon guidance (GO:0031290, BP)	5.4	0.03	Bmpr1b, Ephb2, Epha7, Isl1
Ionotropic glutamate receptor complex (GO:0008328, CC)	5.3	0.04	Gria1, Grin1, Grik5, Dlg4
Histone-lysine N-methyltransferase activity (GO:0018024, MF)	5.0	0.002	Kmt2b, Nsd1, Whsc1, Setdb1, Ehmt1, Suv420h2, Setd7
Regulation of long-term neuronal synaptic plasticity (GO:0048169, BP)	5.0	0.02	Ephb2, Rims1, Grin1, Dlg4, Nras
Cerebral cortex cell migration (GO:0021795, BP)	4.8	0.05	Reln, Psen1, Egfr, Lrp8
Cell adhesion molecule binding (GO:0050839, MF)	4.7	0.05	Psen1, Neo1, Ctnna2, Mllt4
Neuropeptide receptor activity (GO:0008188, MF)	3.9	0.004	Npffr2, Prlhr, Galr1, C130060K24Rik, Npffr1, Ntsr2, Npy1r, Npy5r
Regulation of axonogenesis (GO:0050770, BP)	3.9	0.004	Mapt, Ephb2, Lrrc4c, Psen1, Plxna3, Grin1, Plxnb1, Cacna1a
Cellular response to extracellular stimulus (GO:0031668, BP)	3.8	0.002	Eif2ak4, Tnrc6a, Angptl4, Fos, Psen1, Ppan, Atg16l1, Atf6b, Slc1a2
PDZ domain binding (GO:0030165, MF)	3.7	0.04	Atp2b2, Lpar2, Grik5, Kirrel3, Igsf5
Protein amino acid glycosylation (GO:0006486, BP)	3.6	1.8E-5	St8sia2, Pomt2, B3gnt6, Slc4a10, Rpn2, B3galt1, Dolpp1, Fut8, St6galnac3, Trak1, Psen1, B3gnt5, B3galnt1, Rpn1, B3gnt9, St8sia6, St6gal2
Nuclear chromatin (GO:0000790, CC)	3.4	0.003	Phc1, Dnmt3a, Pcgf2, Sirt7, Hira, Akap8, H2afy2, Smarcb1, Zfp57, Tcf3
Transmembrane receptor protein tyrosine kinase activity (GO:0004714, MF)	3.3	0.003	Pcgf2, Ephb2, Epha7, Epha10, Epha6, Alk, Egfr, Epha8, Kit, Igf1r
Histone acetylation (GO:0016573, BP)	3.3	0.03	Pcgf2, Kat6a, Ing5, Ep300, Mbd3, Tcf3
Neurotransmitter secretion (GO:0007269, BP)	3.2	0.04	Cacna1b, Lin7c, Psen1, Cacna1a, Unc13b, Sv2b
Glycoprotein biosynthetic process (GO:0009101, BP)	3.1	3.7E-5	St8sia2, Pomt2, B3gnt6, Slc4a10, Rpn2, Large, B3galt1, Dolpp1, Fut8, St6galnac3, Trak1, Psen1, Ext1, B3gnt5, B3galnt1, Rpn1, B3gnt9, St8sia6, St6gal2

Shown are the non-redundant GO-terms with numerical identifiers and the domains Biological Process (BP), Molecular Function (MF) or Cellular Component (CC), the fold enrichment (F.E.), p-value and the included genes.

**Table 5:** **Most significantly enriched GO-terms from the genes down-regulated during maturation of the SCG neurons**.

**GO-term**	**F.E**.	***p***	**Genes**
DNA replication, synthesis of RNA primer (GO:0006269, BP)	12.4	0.02	Ccdc111, Helb, Prim1
Glycolipid catabolic process (GO:0019377, BP)	11.1	0.004	Hexa, Hexb, Galc, Gla
Arylsulfatase activity (GO:0004065, MF)	9.8	0.03	Arsb, Arsg, Sulf2
Phosphatidylinositol metabolic process (GO:0046488, BP)	6.0	0.02	Cd81, Pip4k2c, Pip4k2a, Pip5k1b
Nucleotide kinase activity (GO:0019201, MF)	5.8	0.009	Ak5, Mpp5, Ak8, Ak1, Cmpk1
Branched chain family amino acid metabolic process (GO:0009081, BP)	5.5	0.01	Auh, Bckdha, Ghr, Aldh6a1, Acad8
Oxidoreductase activity, acting on the CH-CH group of donors, NAD or NADP as acceptor (GO:0016628, MF)	5.4	0.01	Decr1, Pecr, Blvrb, Dpyd, Ptgr1
Aromatic compound biosynthetic process (GO:0019438, BP)	5.2	0.01	Pts, Mocs1, Spr, Gch1, Pcbd1
Protein homotetramerization (GO:0051289, BP)	5.1	0.04	H2-D1, Hprt, Gpx3, Pcbd1
Palmitoyltransferase activity (GO:0016409, MF)	4.8	0.02	Cpt1a, Cpt1c, Cpt2, Zdhhc1, Zdhhc15
Glycolipid metabolic process (GO:0006664, BP)	4.5	0.009	Hexa, Hexb, Galc, Gla, St6galnac6, Map7
Dopamine metabolic process (GO:0042417, BP)	4.4	0.02	Sncb, Park2, Hprt, Spr, Nr4a2
Purine nucleoside metabolic process (GO:0042278, BP)	4.1	0.01	Pcmt1, Hprt, Nudt7, Mat2b, Ocln, Ppcdc
Acetyl-CoA catabolic process (GO:0046356, BP)	4.1	0.01	Mdh1, Sdha, Nudt7, 2610507B11Rik, Sucla2, Idh3b
Negative regulation of DNA metabolic process (GO:0051053, BP)	4.1	0.03	Ndfip1, Msh6, Brca2, Bcl6, Msh2

Shown are the non-redundant GO-terms with numerical identifiers and the domains Biological Process (BP), Molecular Function (MF) or Cellular Component (CC), the fold enrichment (F.E.), p-value and the included genes.

The GO-terms related to epigenetic repression of the genome and transcription were among the most significantly up-regulated ones, including the genes involved in chromosome condensation (*Akap8, Cdca5*), nuclear chromatin (*Phc1, Dnmt3a, Pcgf2, Sirt7, Hira, H2afy2, Zfp57*), histone acetylation (*Pcgf2, Kat6a*) and histone methyltransferases (*Kmt2b, Nsd1, Whsc1, Setdb1, Ehmt1, Suv420h2*). However, some genes related to transcriptional repression (histone deacetylases *Hdac8, Hdac9, Hdac11*) were down-regulated and some transcriptional activators (*Setd7, Kat6a, Ing5, Ep300*) up-regulated.

Functional categories related to cell surface receptors (several Eph receptors, *Alk, Egfr, Igf1r*) and their signaling (cellular response to extracellular stimulus, transmembrane receptor protein tyrosine kinase activity, PZD domain binding) were also significantly enriched among the up-regulated genes. Also, the cell adhesion-related GO-terms were up-regulated during maturation, including the genes *Psen1* (presenilin 1), *Neo1* (neogenin1), *Ctnna2* (catenin, alpha2), *Mllt4, Reln* (reelin). In contrast, many down-regulated GO-terms appeared to be involved in the various metabolic and biosynthetic processes (Table 5).

### Analysis of the cell death- and survival-related genes

The ending of PCD is a remarkable event during maturation of the SCG neurons. It is genetically determined (Easton et al., 1997; Francis and Landis, 1999; Putcha et al., 2000; Glebova and Ginty, 2005; Young et al., 2011) but very little is known about its mechanisms and the involved genes (Kole et al., 2011). We therefore paid special attention to the changes in the genes related to cell death and survival. Gene ontology cluster analysis (Supplementary file S1) revealed the GO-terms related to apoptosis and cell death among the down-regulated genes (Table 6) but not among up-regulated genes.

**Table 6:** **GO-terms related to cell death or apoptosis that were significantly down-regulated during maturation of the SCG neurons**.

**GO-term**	**F.E**.	***p***	**Genes**
Death (GO:0016265, BP)	1.7	3.7E-4	Fkbp8, Cdip1, Slk, Xaf1, 4632434I11Rik, Ggct, Rassf5, Spr, Msh2, Cyfip2, Cib1, Gatad2a, Lrdd, Asah2, Bag3, Itgb3bp, Gan, Pura, Naip2, Prune2, Bnip3l, Sqstm1, Eda2r, Gulp1, Unc5a, Bcap29, Ypel3, Tax1bp1, Perp, Unc5b, Bcl2l13, Itm2b, Cflar, Rad21, Aktip, Hprt, Nr4a2, Rnf144b, Phlda3, Trp53inp1, Ift57, Ebag9, Wwox, Zmat3, Mef2a, Faim2, Ppm1f, Triap1, Ppt1, Stk3, Tnfrsf10b, Pea15a
Programmed cell death (GO:0012501, BP)	1.7	4.9E-4	
Cell death (GO:0008219, BP)	1.6	6.9E-4	
Apoptosis (GO:0006915, BP)	1.6	0.001	
Regulation of apoptosis (GO:0042981, BP)	1.5	0.003	Msh6, S100b, Cdip1, Scg2, Eef1a2, Bcl6, Wrn, Nol3, Ddit3, Foxo1, Cdkn1a, Msh2, Sncb, Zak, Mitf, Bag3, Aifm2, Rarb, Glo1, Brca2, Snai2, Bnip3l, Tax1bp1, Perp, Gch1, Apoe, Serinc3, Bcl2l13, Itm2b, Adora1, Cflar, Efhc1, Gal, Bmp7, Nr4a2, Sycp3, Phlda3, Trp53inp1, Ift57, Jmy, Wwox, Nr3c1, Sgk3, Xrcc4, Triap1, Ppt1, Stk3, Gfral, Xrcc5, Tnfrsf10b, Pea15a
Regulation of programmed cell death (GO:0043067, BP)	1.5	0.003	
Regulation of cell death (GO:0010941, BP)	1.5	0.003	
Positive regulation of programmed cell death (GO:0043068, BP)	1.6	0.03	
Positive regulation of cell death (GO:0010942, BP)	1.6	0.03	
Negative regulation of apoptosis (GO:0043066, BP)	1.6	0.03	
Negative regulation of programmed cell death (GO:0043069, BP)	1.6	0.04	
Anti-apoptosis (GO:0006916, BP)	2.1	0.04	
Negative regulation of cell death (GO:0060548, BP)	1.6	0.04	
Positive regulation of apoptosis (GO:0043065, BP)	1.5	0.04	

Shown are the GO-terms with numerical identifiers, all belonging to the domain Biological Process (BP), the fold enrichment (F.E.) and the p-value. The indicated genes are associated with all respective GO-terms, albeit at different extent.

We analyzed changes in the genes known to be essential in the survival or death of the sympathetic or other neurons. Only few of those were significantly changed. Genes for the neurotrophic factors related to sympathetic neurons *Ngf*, *Bdnf*, *Ntf3* (NT3), *Gdnf*, *Nrtn, Artn* and their respective receptors *Ntrk2* (TrkB), *Ntrk3* (TrkC), *Ret, Ngfr* (p75^NTR^), *Gfra1, Gfra1*, and *Gfra3* were not significantly changed during neuronal maturation. Surprisingly, the gene for NGF receptor *Ntrk1* (TrkA) was missing from the array. However, activated (phosphorylated) TrkA was shown to be more stable in the mature than immature sympathetic neurons (Tsui-Pierchala and Ginty, 1999) that could contribute to the insensitivity of mature neurons to NGF for survival. We therefore checked the genes reported to be associated with TrkA, such as *Appl1* and *Gipc1* (Lin et al., 2006), Slc9a5 (Diering et al., 2013), *Ccm2* (Harel et al., 2009), *Stk25* (Costa et al., 2012), *Kidins220* (ARMS) (Sniderhan et al., 2008), *Ptpn6* (SHP1) (Marsh et al., 2003), *Jakmip2* (Necc2) (Díaz-Ruiz et al., 2013), *Pincher* (Shao et al., 2002), and *Cox* (cytochrome oxidase) genes (Vaughn and Deshmukh, 2008). All these genes were not significantly changed, suggesting that regulation of the activity of TrkA could not be the main cause why the mature neurons survive without NGF. However, the levels of *Ptpn11*, encoding for the phosphatase SHP2 and reported to regulate positively the signaling of TrkB receptors (Araki et al., 2000; Takai et al., 2002), had slightly increased in the mature neurons (Table 4). The genes reported to interact with p75^NTR^ (Roux and Barker, 2002) such as *Sort1* (sortilin), *Traf6, Maged1* (NRAGE), *Zfp110* (NRIF), *Ngfrap1* (NADE), and *Ndn* (Necdin) were also not significantly changed. In conclusion, the independence of the mature SCG neurons from NTFs for survival seems not to be caused by changes in the levels of NTFs, their receptors and known receptor-interacting proteins.

Among the genes for the core apoptotic machinery proteins, including Bcl-2 family members, caspases, Birc (IAP) family members, but also death receptors, their ligands and the proteins of death-inducing signaling complex (DISC) only few changed significantly upon neuronal maturation (Table 7). The proapoptotic protein Bax is critical for the PCD of SCG neurons (Putcha et al., 1999), as in its absence or upon its blockage the neurons do not die by NGF deprivation (Deckwerth et al., 1996; Aalto et al., 2007). As Bax protein is present but not activated by NGF deprivation in the mature SCG neurons (Easton et al., 1997; Putcha et al., 2000; Kole et al., 2011) we analyzed the reported genes for Bax-interacting proteins, such as *Xrcc6* (Ku70) (Sawada et al., 2003), *mt-Rnr2* (humanin) (Guo et al., 2003), all *Ywha* (14-3-3) genes (Samuel et al., 2001), *Sh3glb1* (Bif-1, endophilin B1) (Wang et al., 2014), *Pin1* (Shen et al., 2009), *Clu* (clusterin) (Zhang et al., 2005), *Npm1* (nucleophosmin) (Kerr et al., 2007), *Cfl1* (cofilin) (Simonishvili et al., 2013), *Tmbim* genes (Rojas-Rivera and Hetz, 2014), and *Nelfb* (Cobra-1) (Ichim et al., 2013) and found that these were not significantly changed during neuronal maturation (except slight increase in *Tmbim1*). Only few genes for classical cytoplasmic kinases whose activity could promote cell survival changed significantly during neuronal maturation (Table 7), whereas, e.g., *Map3k11* (MLK3), *Mapk8* (Jnk1), *Mapk9* (Jnk2), *Akt1*, and *Akt2*, but also phosphatase *Dusp1* (Mkp1) (Kristiansen et al., 2010) remained unchanged. Thus, the levels of the members of core apoptotic machinery or classical survival-promoting kinases are not drastically changed during SCG maturation. The mRNA levels of several transcription factors reported to be related to neuronal maturation or apoptosis, such as *Trp73* (p73) (Walsh et al., 2004), *Trp53* (p53) (Vaughn and Deshmukh, 2007), *Jun* (Easton et al., 1997; Ham et al., 2000) did not change significantly upon neuronal maturation. However, *Nr1d1* (Chomez et al., 2000), *Bcl6* (Otaki et al., 2010), *Foxo1* (Gilley et al., 2003), and *Klf9* (Lebrun et al., 2013) transcripts were all significantly up-regulated in the mature neurons.

**Table 7:** **Genes related to apoptosis or cell survival that changed significantly during SCG neuronal maturation**.

**Gene ID**	**Description**	**log FC**	**adj *p*-value**	**References**
Tnfrsf10b	Tumor necrosis factor receptor superfamily, member 10b [TRAIL-R2/DR5]	−2.29	0.00013	Kichev et al., 2014
Faim2	Fas apoptotic inhibitory molecule 2	−0.84	0.0006	Segura et al., 2007; Yu et al., 2008
Traf4	TNF receptor associated factor 4	1.06	0.0014	
Traf7	TNF receptor-associated factor 7	0.51	0.005	
Bak1	BCL2-antagonist/killer 1 [N-Bak]	0.35	0.032	Sun et al., 2001; Jakobson et al., 2012, 2013
Bcl2l13	BCL2-like 13 (apoptosis facilitator) [Bcl-rambo]	−0.25	0.042	Kataoka et al., 2001
Bnip3l	BCL2/adenovirus E1B interacting protein 3-like [Nix]	−0.39	0.033	Zhang and Ney, 2009
Xaf1	XIAP associated factor 1	−0.71	0.0007	Perrelet et al., 2004
Aifm2	Apoptosis-inducing factor, mitochondrion-associated 2	−0.25	0.049	Hangen et al., 2010
Akt3	Thymoma viral proto-oncogene 3	−0.42	0.0031	
Mapk7	Mitogen-activated protein kinase 7 [Erk5]	0.51	0.0091	
Mapk6	Mitogen-activated protein kinase 6 [Erk3]	0.45	0.014	
Mapk3	Mitogen-activated protein kinase 3 [Erk1]	−0.27	0.036	
Ptpn11	Protein tyrosine phosphatase, non-receptor type 11 [SHP2]	−0.45	0.010	Araki et al., 2000
Klf9	Krüppel-like factor 9	−1.23	0.0017	Lebrun et al., 2013
Nr1d1	Nuclear receptor subfamily 1, group D, member 1	−1.04	0.0002	Chomez et al., 2000
Bcl6	B cell leukemia/lymphoma 6	−1.11	0.0002	Otaki et al., 2010
Foxo1	Forkhead box O1	−0.88	0.003	Gilley et al., 2003
Tmbim1	Transmembrane BAX inhibitor motif containing 1	−0.71	0.002	Rojas-Rivera and Hetz, 2014

We were surprised to find that core apoptotic machinery was basically not changed in the neurons whose PCD was over. To confirm this finding we analyzed the array data also with Gene Set Enrichment Analysis (GSEA) another web-based method that uses the whole gene sets to interpret the array data and is therefore useful to test specific hypotheses (Supplementary files S3, S4). No gene sets related to apoptosis and cell death were revealed in the young neurons, whereas 11 were found in the mature neurons that, however had very high normalized *p*-values and FDR's (Supplementary file S4) and are most likely false positives. Thus, also GSEA did not reveal significant changes in the classical apoptotic genes in the mature neurons.

### qPCR analysis of selected genes in the immature and mature sympathetic ganglia isolated from mouse and rat

As an example of the usage of array data we selected five genes of interest: *Igf2bp3, Coro1A, Zfp57, Dcx*, and *Apaf* (Table 8) that could potentially be involved in the neuronal maturation and whose expression changes were also revealed by the microarray. Expression of these genes was analyzed by qPCR in the immature and mature ganglia from two species—mouse and rat. To gain insight whether gene expression changes obtained from *in vitro* cultures reflect the situation *in vivo*, total RNA from freshly prepared whole ganglia from the newborn, 2 and 3 weeks old mice and rats were analyzed. The data were normalized to the *Nefh* (neurofilament heavy polypeptide) gene that is expressed only in the neurons and whose expression level did not change significantly in the SCG during the analyzed time points (see the Materials and Methods).

**Table 8:** **Genes selected from the microarray for qPCR analysis**.

**Gene ID**	**Description**	**log FC**	**adj *p*-value**
Igf2bp3	Insulin-like growth factor 2 mRNA binding protein 3	1.81	0.000828
Zfp57	Zinc finger protein 57	1.94	0.052627
Dcx	Doublecortin	2.10	0.000497
Coro1a	Coronin, actin binding protein 1A	0.93	0.052746
Apaf1	apoptotic peptidase activating factor 1	0.71	0.095022

**Figure 3:**
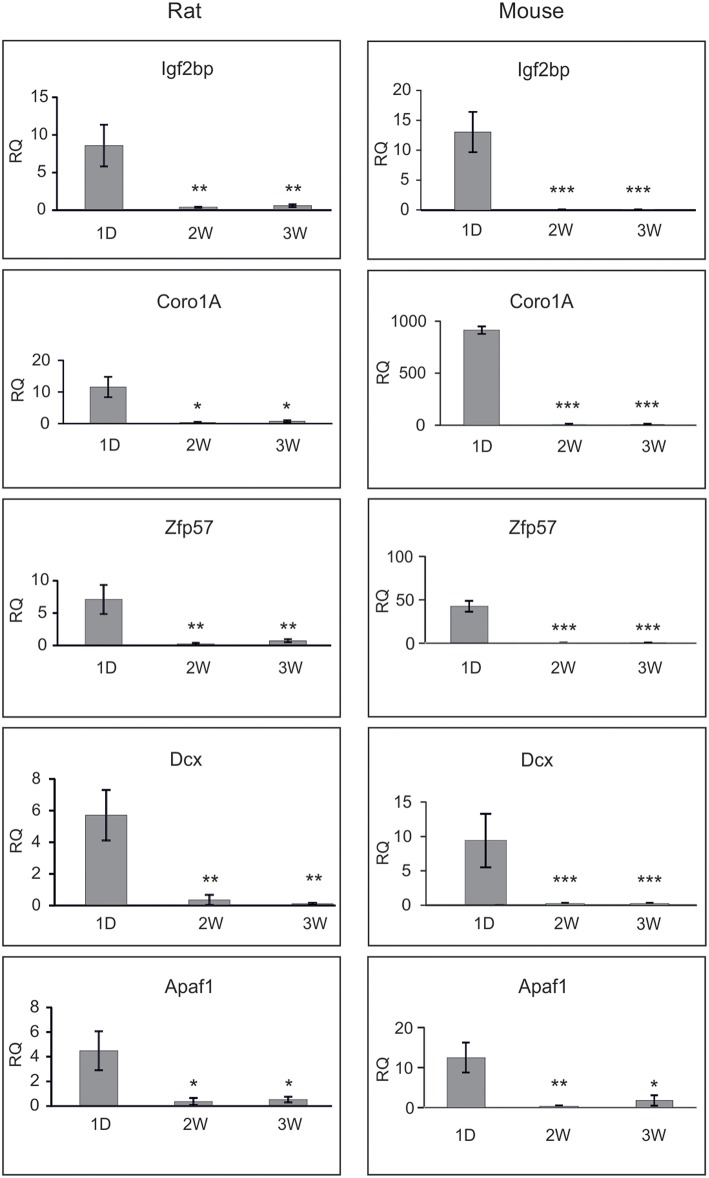
**qPCR analysis of the selected genes during maturation of rat SCG**. Shown are the relative mRNA quantifies (RQ) from newborn (1D), 2 weeks (2W) and 3 weeks (3W) old rat and mouse SCG, normalized to the levels of *Nefh* and calibrated against the laboratory golden standard. Three independent analyses (except four for rat *Apaf1*) were performed and the means were compared with one-way ANOVA followed by Tukey-Kramer Multiple Comparisons Test. Shown are the means ± S.E.M. ^*^p < 0.05; ^**^p > 0.01, ^***^p > 0.001. The null hypothesis was rejected at *p* < 0.05.

The results are presented on Figure 3. The mRNAs for *Igf2bp3, Coro1A, Zpf57*, and *Dcx* were drastically reduced by second week of postnatal development in both animal species, and very low levels of these genes persisted by third postnatal week. All these genes were also significantly down-regulated in the microarray assay (Table 8). *Apaf1* mRNA was also significantly down-regulated in the mature neurons as reported by others (Wright et al., 2007), although the change was less significant in the microarray. Thus, the changes in these selected genes determined by microarray in the cultured neurons mostly matched the changes determined by qPCR from the whole ganglia.

## Discussion

In this study we compared the gene expression profiles of immature and mature sympathetic neurons to get first insight of the poorly studied processes of maturation, including the control of PCD (Kole et al., 2013; Lebrun et al., 2013). To our knowledge, the current analysis is the first one performed on the immature and mature SCG neurons. Many changes that occur during neuronal maturation are permanent, suggesting stable changes in the gene expression. Therefore, our analysis, deposited to the public database, could be a valuable source in the studies of neuronal maturation.

The majority of mouse and rat SCG neurons exit the cell cycle before birth (Hendry, 1977; Shi et al., 2008). Thus, in the neonatal mouse ganglia most of the dividing cells are glial progenitors that give rise to the satellite cells, except a small population of the neurons that became postmitotic during first postnatal week (Shi et al., 2008). The neuritogenesis, target innervation and synaptogenesis of the sympathetic neurons also begin in the embryogenesis but occur mostly during postnatal maturation (De Champlain et al., 1970; Schotzinger and Landis, 1990). This correlates well with our GO analysis showing enrichment of the GO terms related to neurite maturation, synaptic plasticity, neuronal migration, ion channels and neurotransmitter receptors in the mature neurons. Also, the main known markers for the sympathetic neurons (*th, ddc, dbh, npy, Eno2, Slc18a2*) did not change significantly during maturation, showing that the basic sympathetic phenotype was already established in the mice by birth.

Many GO-terms concerning the modification of chromatin and regulation of transcription were significantly up-regulated showing that the postnatal maturation of the sympathetic ganglia involves epigenetic regulation of gene expression that could be the last steps of terminal differentiation. Most of the genes in these GO-terms have been related to chromatin condensation and gene repression, suggesting that in the mature neurons, chromatin is more repressed than in the immature ones. However, some transcriptional repressors were down-regulated and some transcriptional activators up-regulated, suggesting that some genes are also activated during maturation. The GO-terms related to cell surface receptors, their signaling and cell adhesion molecules were also significantly enriched in the mature neurons, suggesting that extracellular signaling and increased cell adhesion play important role in the maturation of SCG neurons. The most significantly down-regulated GO-terms included genes that are related to the metabolic and biosynthetic processes. It is difficult to conclude from the GO-terms the details of the metabolic changes and we therefore conclude only that the metabolic activity is high in the neonatal SCG neurons but could decrease during their maturation.

The end of PCD is a remarkable event in the postnatal maturation of the SCG neurons that occurs largely via intrinsically determined genetic events rather than the extracellular signals, as it appears in the dissociated cultures with similar time schedule as in the whole ganglia *in vivo* (Easton et al., 1997; Francis and Landis, 1999; Putcha et al., 2000; Glebova and Ginty, 2005; Young et al., 2011). We therefore paid special attention to the genes related to cell survival and death. Individual checking of the classical genes for apoptosis or cell survival did not reveal major changes in their levels during neuronal maturation, except *Apaf1* that was clearly reduced, as also reported by others (Wright et al., 2007). This conclusion was supported by alternative GSEA method that also did not reveal significant changes in the apoptotic and cell death related gene sets. Active killing of the neurons via neurotrophin receptor p75^NTR^ (Deppmann et al., 2008; Hempstead, 2014) or dependence receptors such as Ret (Bordeaux et al., 2000) or TrkC (Tauszig-Delamasure et al., 2007) but also TrkA (Nikoletopoulou et al., 2010) could contribute to neuronal PCD. However, these receptors and the proteins known to associate with them were not significantly down-regulated in the mature SCG neurons and might not be the major mechanism of the NGF-independency. Low levels of *Apaf1* (Wright et al., 2007), this study, and repression of transcripts for some Bax-activating BH3-only proteins (Bim, DP5/Hrk, PUMA) by microRNAs in the mature SCG neurons (Kole et al., 2011) certainly contribute to their independence of NGF for survival. However, additional yet to be discovered genes/proteins could be involved in the termination of PCD that is a major process crucially changing the status of the neuron (Kole et al., 2013). Our cluster analysis revealed significant enrichment of the GO-terms related to cell death and apoptosis among down-regulated genes, but not up-regulated genes, suggesting general suppression of the apoptotic program. Notably the apoptotic functions of most of the genes associated with these GO-terms are currently poorly characterized. We propose that the end of PCD is mediated by yet to be found genes whose products control the activity of apoptotic machinery.

Our microarray data offer a good choice to select the candidate genes for the further studies of neuronal maturation. In this study we selected some genes of interest and studied their expression changes in the immature and mature SCG by qPCR. The selected genes changed significantly during neuronal maturation, were revealed by the gene ontology analysis and could potentially participate in this process, but their role in the cell death and neuronal maturation is currently poorly studied. The genes were analyzed *in vivo* by using neuronal marker gene *Nefh* as a reference to overcome the confounding effect of non-neuronal cells present in the ganglia but mainly removed from the neuronal cultures used for the microarray analysis. The expression changes of the selected genes analyzed from the *in vitro* cultures by microassay and from the whole ganglia by qPCR principally matched. We want to stress, however, that such match is not quaranteed, as the culture conditions may change gene expression. E.g., much smaller down-regulation of the Apaf1 gene was revealed by microarray than by qPCR in our study. How the culture conditions affect gene expression in the SCG neurons is currently poorly known and warrants further studies.

Igf2bp3 (insulin-like growth factor 2 mRNA binding protein 3) is an RNA-binding protein, involved in the RNA synthesis, metabolism and stability in different cells including the neurons (Mori et al., 2001). Coro1A, a cytoskeleton binding protein, was shown to bind and regulate TrkA-NGF-containing signaling endosome and thereby increase the survival of the SCG neurons (Suo et al., 2014). We analyzed Dcx (doublecortin), a microtubule-associated protein expressed by migrating immature neurons but down-regulated during neuronal maturation (Francis et al., 1999; Gleeson et al., 1999), and found that it was down-regulated also in the mature SCG. Down-regulation of the mRNA for Apaf1 (apoptotic peptidase activating factor 1) in the mature SCG (Wright et al., 2007) was confirmed here as well. Two transcription factors were selected for further analysis. Zfp57 (zinc finger protein 57) is related to genomic methylation imprinting (Zuo et al., 2012). Our analysis of genes expressed differently in the immature and mature sympathetic neurons could provide a good candidate gene list for further studies addressing the genetic program governing the maturation of neurons.

## Author contributions

MR and JP carried out the preparation of the ganglia and real-time qPCR experiments. ML performed analysis of the array data. UA designed the experiments and wrote the manuscript. All authors read and approved the final manuscript.

### Conflict of interest statement

The authors declare that the research was conducted in the absence of any commercial or financial relationships that could be construed as a potential conflict of interest.

## Abbreviations

NGF: nerve growth factor
SCG: superior cervical ganglion
PCD: programmed cell death
NTF: neurotrophic factor
FDR: false discovery rate
PCA: principle component analysis
GO: gene ontology
RT: reverse transcriptase
qPCR: quantitative polymerase chain reaction
DIV: days *in vitro*.
